# Adjuvant immunotherapy in high-risk stage II melanoma: real-world outcomes from a large multicenter retrospective study

**DOI:** 10.1007/s00262-026-04466-0

**Published:** 2026-07-17

**Authors:** Vincenzo De Falco, Antonino Colloca, Giuseppe Argenziano, Marcella Scala, Michele Guida, Margaret Ottaviano, Maria Chiara Sergi, Mariacarmela Santarpia, Teresa Del Giudice, Antonio Maria Grimaldi, Silvana Cozzolino, Miriam Forte, Francesco Caraglia, Eleonora Cioli, Alfonso Esposito, Maria Cristina Giugliano, Francesca Sparano, Francesco Iovino, Salvatore Tolone, Giovanni Docimo, Giuseppe Colella, Mario Faenza, Renato Franco, Camila Scharf, Anna Russo, Luigi Formisano, Davide Ciardiello, Paolo Antonio Ascierto, Fortunato Ciardiello, Teresa Troiani

**Affiliations:** 1 Department of Precision Medicine, Università Degli Studi Della Campania Luigi Vanvitelli, Via Sergio Pansini 5, 80131 Naples, Italy; 2 Department of Mental and Physical Health and Preventive Medicine, University of Campania Luigi Vanvitelli, 80138 Naples, Italy; 3 Clinical Medicine and Surgery, University of Naples Federico II, Naples, Italy; 4 Istituto Tumori IRCCS Giovanni Paolo II, Bari, Italy; 5 Unit of Melanoma, Cancer Immunotherapy and Development Therapeutics, Istituto Nazionale Tumori IRCCS Fondazione Pascale, Naples, Italy; 6 San Paolo Hospital, Bari, Apulia Italy; 7 Department of Human Pathology “G. Barresi”, University of Messina, 98125 Messina, Italy; 8 Oncology Department, Oncology Unit, “Azienda Ospedaliero Universitaria (AOU) Dulbecco” Hospital, Catanzaro, Italy; 9 Medical Oncology Unit, AORN San Pio, Benevento, Italy; 10 Department of Translational Medical Sciences, University of Campania Luigi Vanvitelli, 80138 Naples, Italy; 11 Multidisciplinary Department of Surgical and Dental Sciences, University of Campania Luigi Vanvitelli, 80138 Naples, Italy; 12 Division of Gastrointestinal Medical Oncology and Neuroendocrine Tumors, European Institute of Oncology, IRCCS, Milan, Italy

**Keywords:** Cutaneous Melanoma, Adjuvant Setting, Anti-PD-1, Adverse Events, Real-World, Stage IIB-IIC

## Abstract

**Background:**

Cutaneous melanoma (CM) is an aggressive cancer since early stages. Stage IIB/C CM present significant recurrence risk. Recently, adjuvant immunotherapy demonstrated to increase recurrence-free survival (RFS) in this setting.

**Objective:**

Assess safety and efficacy of adjuvant immunotherapy for stage IIB/C CM in a real-world setting.

**Methods:**

Stage IIB/C CM patients treated with adjuvant immunotherapy in eight centers from the “Gruppo Oncologico Italia Meridionale” (GOIM) were retrospectively investigated. Data were collected over a median follow-up of 16 months.

**Results:**

Ninety-six patients with a median age of 59 years were included. Most participants were male (64.6%). Approximately 60.4% were classified stage IIB and 39.6% stage IIC, with the majority of tumors localized on the trunk (54.2%). Toxicity occurred in 63.5% of patients with 79.7% of these events classified as grade 1–2 (CTCAE v5.0). The most reported events were thyroid dysfunction, fatigue, gastrointestinal disorders and itchiness. Recurrence was observed only in 7 patients (7.3%).

**Conclusions:**

The 12-month and 24-month RFS were respectively 98.7% and 82.6%. The toxicity profile was generally manageable in a real-life population, however 17.6% of all patients experienced grade 3–4 AEs, underscoring the importance of evaluating adjuvant therapy in a multidisciplinary setting.

**Supplementary Information:**

The online version contains supplementary material available at https://doi.org/10.1007/s00262-026-04466-0 .

## Introduction

Cutaneous melanoma (CM) is a highly aggressive malignancy arising from melanocytes, characterized, even at early stage, by a significantly higher metastatic potential compared with most solid tumors [1]. Wide local excision with adequate margin, eventually together with sentinel lymph node biopsy (SLNB) is the standard of care in patients without distant metastases [2, 3]. Further treatments, including loco-regional and systemic treatments, are guided by disease stage and molecular characteristics [4]. Recently, different studies evaluated the advantages of adjuvant treatment to reduce the risk of recurrence in non-metastatic CM after complete excision [5]. Adjuvant therapies for stage III melanoma with immune checkpoint inhibitors (Pembrolizumab and Nivolumab) or targeted therapy (Dabrafenib and Trametinib, only for BRAFV600 mutated melanoma) are already well-established [5–8]. Melanoma patients with a Breslow depth > 4 mm or ulcerated tumor with a Breslow depth > 2.1 mm (stage IIB and IIC respectively) in absence of nodal metastases, are associated with lower melanoma-specific overall survival than stage IIIA, thereby supporting recent efforts to assess adjuvant therapeutic strategies [9, 10]. In fact, two phase III trials, KEYNOTE-716 and CheckMate 76 K, randomized patients with stage IIB or IIC melanoma to receive one year of pembrolizumab or nivolumab, respectively, versus placebo. Both trials demonstrated a statistically significant increase of recurrence-free survival (RFS) and distant metastasis free survival (DMFS) and therefore both pembrolizumab and nivolumab have been approved in several countries, including Italy [11–13]. On the other hand, 8 out of 10 patients in both studies experienced AEs, about 15% of which G3-G4 [11–13]. In addition, around 15% of patients experienced endocrine dysfunction which could possibly lead to a permanent hormonal replacement therapy, with high impact on patient’s QoL [11–13].

However, real-world data are lacking, preventing a comprehensive understanding of the treatment’s effectiveness and of its risk–benefit profile outside of controlled clinical trials. In this context, our multicentric study aims to provide the first real-world evidence regarding the effectiveness and safety of adjuvant immunotherapy in high-risk stage II CM.

## Patients and methods

Clinical data from patients affected by stage IIB and IIC melanoma treated with adjuvant anti-PD-1 therapy in eight centers in Southern Italy, within the Gruppo Oncologico dell’Italia Meridionale (GOIM), were retrospectively collected and investigated.

Inclusion criteria were: adult patients (age > 18 years) with a histological diagnosis of stage IIB and IIC cutaneous melanoma treated with adjuvant immunotherapy (Pembrolizumab or Nivolumab) for at least 1 cycle. All patients meeting these criteria were included in the study, no patients were excluded due to missing clinical data, and none were lost to follow-up at the time of data cut-off. Data were collected from July 2023 to October 2025. Patients who underwent adjuvant immunotherapy from July 2023 to October 2025 were investigated, the enrollement flowchart has been provided as Supplementary material (Supplementary Fig. 1).

During adjuvant therapy, as for Italian follow-up guidelines, patients underwent total-body computed tomography (CT) scans every 6 months to assess disease recurrence, as well as nodal basin ultrasonography every 3 months. After completion of adjuvant treatment, patients continued to be followed with the same imaging schedule, in order to monitor for late recurrences. Clinical evaluation was performed concomitantly with imaging assessments throughout the entire follow-up period. Patients affected by concomitant malignancies, except for non-melanoma skin cancers or tumors in situ or benign neoplasms, were excluded. Patients demographics, clinical and pathological characteristics, as well as tumor responses and survival outcomes were collected. The study protocol approval was provided by the Institutional Review Board of the University of Campania and conducted in compliance with the Declaration of Helsinki and Good Clinical Practice (GCP) guidelines. Written informed consent from each patient was collected, authorizing the use of anonymized data for research purposes. Adverse events were recorded and graded based on the National Cancer Institute Common Terminology Criteria for Adverse Events (NCI CTCAE), version 5.0. RFS curves were estimated using the Kaplan–Meier method. GraphPad (version 9.0.0) and MedCalc (Ver 23.1.3) were used to generate survival curves and to calculate statistical data. Statistical significance of survival curves was assessed using the Log-rank test. To further investigate the association between baseline clinicopathological variables and RFS, Cox proportional hazards models were performed. Univariable Cox analyses were conducted for age, sex, primary tumor site, ulceration status, stage, and BRAF mutation status. Given the limited number of recurrence events, multivariable analyses were restricted to parsimonious models to reduce the risk of overfitting. Specifically, adjusted Cox models including age, sex, and one additional clinically relevant variable at a time were performed. Due to the presence of sparse-event categories and the small number of events, standard Cox regression models yielded unstable estimates for some variables. Therefore, penalized Cox regression using Firth’s correction was additionally performed as a sensitivity analysis. All statistical analyses were performed using R version 4.5.2 (2025–10-31). A two-sided *p*-value < 0.05 was considered statistically significant. ESMO GROW Checklist has been assessed to ensure an adequate reporting of real-world data.

## Results

### Patients characteristics

Between July 2023 and October 2025, data were collected from 96 patients with stage IIB and IIC melanoma who received adjuvant immunotherapy. As a result of the delayed approval of nivolumab for this indication, all patients in this study received pembrolizumab treatment. The median age at the diagnosis was 59 years, ranging from 32 to 85 years. Overall, a predominance of male patients was observed (64.6%) (Table 1). The most frequent melanoma primary melanoma site was the trunk (54.2% of cases), followed by the lower limbs (21.9%), head and neck region (15.6%) and upper limbs (8.3%) (Table 1). Ulceration was reported in 83.3% of cases analyzed. Approximately 43.7% of tumors were classified as pT3b and 16.7% as pT4a, with the remaining 39.6% corresponding to stage IIC (pT4b) (Table 1). Consistent with standard clinical practice, lymph node biopsy was performed in the majority of patients (86.4%); however, 13.5% of patients did not undergo the procedure, following tumor board discussion, due to comorbidities or patient refusal. Although molecular profiling was not performed in 53.1% of patients, among patients with available molecular profiling 21.9% harbored *BRAF V600* mutation, while 25% were *BRAF* wild type (WT). *NRAS* mutations were not assessed in 55.2% of patients; however, 7.3% were found to carry NRAS mutations.

**Table 1:** Patient characteristics

Characteristics			Number of patients (%)
Sex	Male		62 (64.6%)
	Female		34 (35.4%)
Median age (range)			59 (32–85)
Primary site	Head/neck		15 (15.6%)
	Trunk		52 (54.2%)
	Upper limbs		8 (8.3%)
	Lower limbs		21 (21.9%)
BRAF status	Wild Type		24 (25%)
	V600		21 (21.9%)
	Unknown		51 (53.1%)
NRAS status	Wild Type		36 (37.5%)
	Mutated		7 (7.3%)
	Unknown		53 (55.2%)
Ulceration	Yes		80 (83.3%)
	No		16 (16.7%)
Sentinel node biopsy	Yes		83 (86.5%)
	No		13 (13.5%)
Disease staging	IIB	T3b	42 (43.7%)
		T4a	16 (16.7%)
	IIC	T4b	38 (39.6%)

### Recurrence free survival

**Fig. 1:**
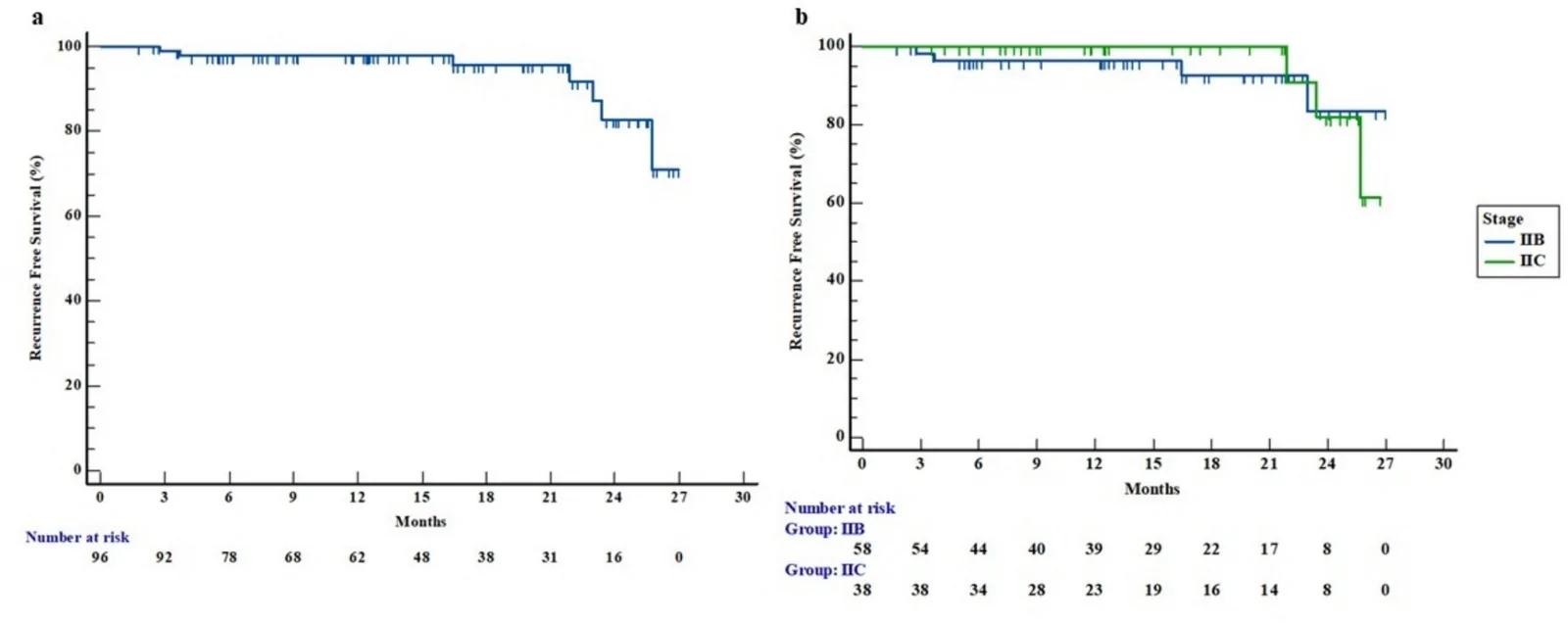
Kaplan Meier survival curve for RFS (**a**) and RFS by melanoma staging (IIB and IIC) (**b**)

As expected, with a median follow-up time of 16 months (range: 2.7–26.9 months), the median RFS for the study cohort was not reached (Fig. 1). At 12 months, the RFS probability was 98.7% (number at risk: 62; SE: 1.5%, IC95%: 0.948–1.000), decreasing to 82.6% at 24 months, considering the 16 months median follow-up period. (number at risk: 16; SE: 7.3%, IC95%: 0.681–0.971) (Fig. 1a). Further analysis revealed no statistically significant difference in RFS between patients with stage IIB and stage IIC disease (*p* = 0.84) (Fig. 1b). Within the follow-up period, recurrence was observed in 7 out of 96 patients (7.3%). In particular, recurrence occurred during immunotherapy in 2 patients (2.1%) while the remaining 5 patients (5.2%) experienced disease recurrence after completion of adjuvant therapy (Table 2). Particularly, recurrence at a loco-regional level (peri-cicatric or nodal) in 4 patients (4.2%) whereas 3 patients (3.1%) developed distant metastases (Table 2). To further explore the association between baseline clinicopathological characteristics and RFS, Cox proportional hazards models were performed. In univariable analysis, stage was not significantly associated with RFS (stage IIC vs. IIB: HR 0.86; 95% CI, 0.19–3.89; *p* = 0.84). Similarly, no significant associations were observed for age (≥ 75 vs. < 75 years: HR, 0.58; 95% CI, 0.07–4.82; *p* = 0.61) or sex (HR, 0.55; 95% CI, 0.12–2.54; *p* = 0.45). Given the limited number of recurrence events (7 events among 96 patients), multivariable analyses were restricted to parsimonious models. In a penalized Cox regression model adjusted for age and sex, stage was not associated with RFS (IIC vs IIB: HR, 0.78; 95% CI, 0.16–3.47; *p* = 0.74). Likewise, no significant associations were observed for age (HR, 0.64; 95% CI, 0.06–3.43; *p* = 0.63) or sex (HR, 0.53; 95% CI, 0.12–2.31; *p* = 0.39). Exploratory analyses including other baseline variables (primary tumor site, ulceration, and BRAF mutation status) were conducted using penalized Cox regression. No statistically significant associations with RFS were observed for ulceration or primary tumor site. BRAF status was evaluated as an exploratory variable using penalized Cox regression. To avoid misclassification bias, BRAF status was first analyzed as a three-category variable, including BRAF-mutated, BRAF wild-type, and unknown status. Compared with BRAF-mutated tumors, BRAF wild-type tumors showed a non-significant trend toward a lower risk of recurrence (HR 0.21, 95% CI 0.02–1.21; *p* = 0.083), whereas patients with unknown BRAF status showed a lower estimated recurrence risk (HR 0.06, 95% CI 0.005–0.42; *p* = 0.004). However, unknown BRAF status represents missing molecular information rather than a biologically defined subgroup and was therefore not considered suitable for biological interpretation.

**Table 2:** Relapse characteristics

Patients recurred at data cut-off	Recurred On immunotherapy	2 (2.1%)
	Recurred Off immunotherapy	5 (5.2%)
	No Recurrence	89 (92.7%)
Pattern of recurrence	Loco-regional	4 (4.2%)
	Distant	3 (3.1%)
	Loco-regional + Distant	0 (0%)

As a sensitivity analysis, patients with unknown BRAF status were excluded. In this restricted cohort, the direction of the association remained consistent, although the association was not statistically significant (BRAF wild-type vs BRAF-mutated: HR 0.28, 95% CI 0.03–1.49; *p* = 0.142). Overall, given the very limited number of recurrence events, the BRAF-related analyses were considered exploratory and interpreted with caution.

In this context, effect estimates may be influenced by sparse data and potential selection bias. Overall, these findings were consistent with the Kaplan–Meier analysis, which did not demonstrate significant differences in RFS according to stage or other baseline characteristics.

### Safety

**Fig. 2:**
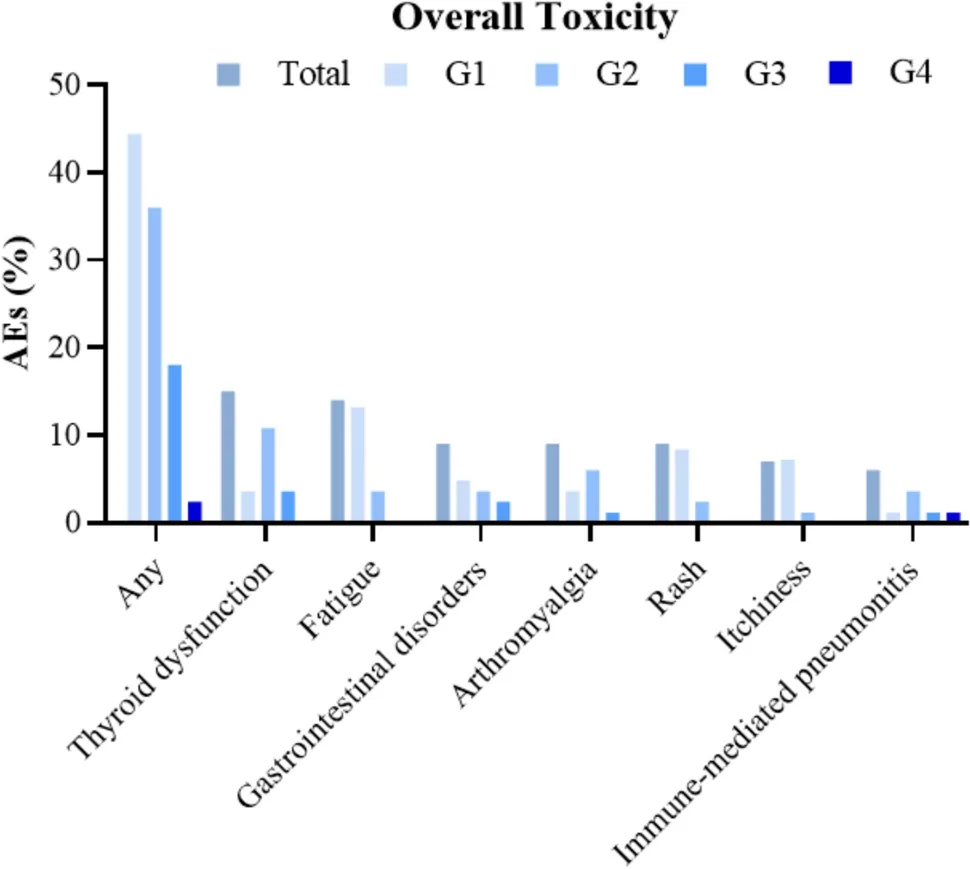
Main Adverse Events (AEs) and their CTCAE grading

Overall, anti-PD-1 therapy demonstrates a manageable safety profile, with a total of 84 adverse events (AEs) reported in 63.5% of patients. CTCAE grading revealed that most AEs were grade 1 and 2, accounting for 44% and 35.7% of all reported toxicities, respectively; on the other hand, among all toxicities reported grade 3 AEs represented 17.9% of cases, while grade 4 events were rare, comprising only 2.4% (Fig. 2, Table 3). Overall, 17.6% of all patients experienced G3-G4 toxicities.

**Table 3:** Adverse Events

Adverse Events	Total	G1—G2	G3—G4
Any	84	67 (79.7%)	17 (20.3%)
Thyroid dysfunction	15 (17.8%)	12 (14.3%)	3 (3.6%)
Fatigue	14 (16.7%)	14 (16.7%)	0 (0%)
Itchiness	7 (8.3%)	7 (8.3%)	0 (0%)
Gastrointestinal disorders	9 (10.7%)	7 (8.3%)	2 (2.4%)
Arthromyalgia	9 (10.7%)	8 (9.5%)	1 (1.2%)
Rash	9 (10.7%)	9 (10.7%)	0 (0%)
Immune-mediated pneumonitis	6 (7.1%)	4 (4.8%)	2 (2.4%)
Increased transaminases	4 (4.8%)	1 (1.2%)	3 (3.6%)
Sicca Syndrome	4 (4.8%)	2 (2.4%)	2 (2.4%)
Pancreatitis	3 (3.6%)	1 (1.2%)	2 (2.4%)
Mucositis	2 (2.4%)	2 (2.4%)	0 (0%)
Cardiotoxicity	1 (1.2%)	0 (0%)	1 (1.2%)
Nephritis	1 (1.2%)	0 (0%)	1 (1.2%)
Patients who experienced toxicities	61 (63.5%)		
Patients who temporarily suspended the treatment due to toxicity	23 (23.9%)		
Patients who definitively discontinued the treatment due to toxicity	16 (16.7%)		
Patients who required corticosteroid treatment	24 (24.4%)		

The most common toxicity experienced was thyroiditis evaluated as hyperthyroidism/hypothyroidism (17.6%), followed by fatigue (16.7%), gastrointestinal disorders (10.7%), arthromyalgia (10.7%), rash (10.7%) (Fig. 2, Table 3). Additionally, 4 patients (4.8%) experienced increased aspartate and alanine transaminases, 3 patients (3.6%) pancreatitis and 2 patients (2.4%) mucositis (Table 3).

Among the less common AEs, 4 patients (4.8%) developed sicca syndrome, presenting with varying degrees of xerostomia and xerophthalmia. These cases required corticosteroid therapy along with supportive care, given the reported impact on quality of life. Notably, one patient (1.2%) developed cardiotoxicity and another (1.2%) experienced nephritis with acute renal insufficiency (Table 3).

Study population included a subset of 19 over-75 years old patients, out of this subgroup 16 (84.2%) patients experienced AEs, with 4 patients (21.1%) experiencing severe G3 AEs, including diarrhea, pneumonitis, nephritis and hepatitis. In contrast, the remaining population under 75 years old presented a reduced incidence of AEs, being experienced in 45 out of 77 patients (58.4%), with severe AEs reported in 13 patients (16.9%).

Temporary treatment interruption was necessary in 23.9% of patients, while permanent discontinuation of therapy occurred in 16.7% of patients. Corticosteroid treatment was required in 24.4% of cases, as per standardized clinical guidelines (Table 3). Lifelong hormone replacement therapy was required in 9.8% of patients, experiencing thyroid dysfunction with a CTCAE Grading ≥ 2. No treatment-related deaths were reported.

### Management of relapse

**Fig. 3:**
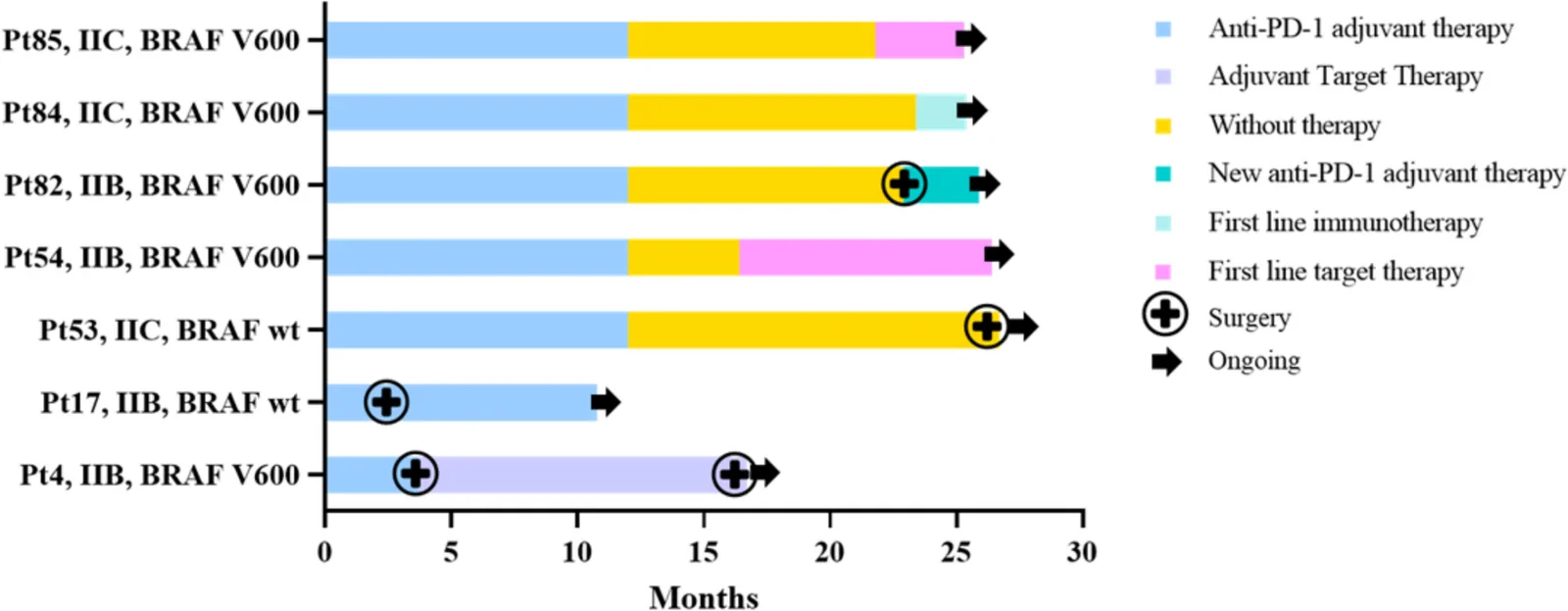
Management of relapsed patients. Pt: Patient. On the Y axis, patients’ basal characteristics have been reported

Overall, 7 patients experienced local or systemic disease relapse. The management of disease recurrence is summarized in Fig. 3. Patient (Pt) 4 and Pt 17 experienced recurrence during anti-PD-1 adjuvant immunotherapy. Pt4 harbored a *BRAF V600* mutation and was subsequently switched to adjuvant targeted therapy, being considered as a stage III disease; following completion of the 1-year adjuvant period, a new peri-cicatricial recurrence was detected and surgically excised. In contrast, Pt17 underwent surgical resection of the regional nodal metastasis and continued adjuvant immunotherapy, which remains ongoing. The remaining five patients experienced recurrence after completing immunotherapy. Pt53 developed a peri-cicatricial recurrence 14.7 months after the of adjuvant immunotherapy, which was surgically removed. Pt82 presented a locoregional nodal relapse 10.9 months post-therapy; the recurrence was surgically excised, and the patient started a new course of anti-PD-1 adjuvant immunotherapy. Finally, Pt54 and Pt84 relapsed with lung metastases at 4.4 months and 11.4 months, respectively, Pt85 relapsed developed nodal metastases 9.8 months after therapy. Pt54 and Pt85 initiated first-line targeted therapy, whereas Pt84 began anti-PD-1 monotherapy as first line treatment. At data cut-off, all patients are alive and, despite the limitations due to data immaturity, were continuing their treatment successfully.

## Discussion

In July 2023, the Italian Medicines Agency (AIFA) approved the first adjuvant anti-PD-1 immunotherapy for stage IIB-C cutaneous melanoma, based on the results of KEYNOTE-716, which demonstrated the efficacy and safety of pembrolizumab in this setting. Subsequently, nivolumab was also approved in the same setting based on the results of CheckMate76K. Despite the positive outcomes reported in pivotal trials, clinical practice often involves variables not reflected in the trial design, which may influence treatment effectiveness. This underscores the importance of real-world evidence studies to validate and complement these findings.

In our real-world study, with a median follow-up of 16 months, median RFS was not reached. The 12- month RFS rate (98.7%) was considerably higher than that reported for the pembrolizumab arm in KEYNOTE-716 trial (90.1%), and the nivolumab arm in the CheckMate76K (89%) [11–13]. Conversely, the 24-month RFS rate (82.6%) was more consistent with the findings of KEYNOTE-716 (81.2%) and CheckMate 76 K (76.5%) [11–13]. The differences observed may be attributed to the smaller sample size and the shorter follow-up period in our study compared to the registrational trials [11–13]. Even more, the results of the 24-months RFS analysis in the present study requires careful interpretation due to the median follow-up of 16 months and the small sample size as it can easily lead to altered estimation of the real RFS value. Also, in the present study 13.5% of patients who did not underwent sentinel node biopsy were included, differing from the standardized eligibility criteria of the registrational studies. As these patients are considered as stage IIB/C MM and candidate to adjuvant immunotherapy, this subpopulation has been maintained to ensure generalizability of the results.

Regarding the toxicity profile, registrational studies reported that in KEYNOTE-716, 82.6% of patients experienced AEs of any grade, with grade 3–4 AEs occurring in 16% of patients [11, 12]. Similarly, in the CheckMate76K trial, 83% of patients reported AEs of any grade, with 14% experiencing grade 3–4 events [13]. In both studies, treatment discontinuation due to unacceptable toxicities occurred in 15% of patients [11–13]. In this study, a comparable proportion of grade 3–4 AEs and treatment discontinuations was observed; however, the overall incidence of AEs of any grade was lower (63.5%). The most frequently reported AEs were thyroid dysfunctions (hyperthyroidism or Hypothyroidism), fatigue and cutaneous reactions or pruritus. These findings align with those reported in the pivotal studies and with other real-world studies in stage III melanoma [11–14]. Particularly, we did not report any cases of adrenal insufficiency which were conversely reported in the registrational studies, however a longer follow-up period could uncover this AE as it can be considered a late-onset toxicity. It should be noted that our retrospective analysis did not account for mean corticosteroid dosages, duration of therapy, or the clinical outcomes of patients experiencing toxicities. Therefore, further research is warranted to clarify how these treatment-related AEs influence the QoL of individuals receiving adjuvant immunotherapy for stage II melanoma.

Overall, our study represents the first retrospective evaluation in literature of the efficacy and safety of immunotherapy in the high-risk stage II melanoma, demonstrating a favorable risk–benefit profile. Nevertheless, longer follow-up and a larger patient cohort are needed to fully assess this aspect. Many questions remain regarding the utility of adjuvant therapy in patients who are often cured by surgery alone. Pivotal studies estimate that 6–8 patients need to be treated to prevent a single recurrence at this stage, highlighting the risk of overtreatment, exposure to potentially lifelong serious adverse events, and increased healthcare costs [15, 16]. In this context, Gawaz’s retrospective analysis about the efficacy of adjuvant anti-PD-1 treatment in melanoma patients ≥ 75 years included a small cohort of stage II patients. The group tolerated treatment well, and there was no discernible variation in the total incidence of AEs. In terms of effectiveness, individuals who were 75 years of age or older had a noticeably lower RFS. In addition to being less efficacious, adjuvant immunotherapy was linked to a higher risk of cutaneous, renal, or intestinal damage in people over 75 and gastrointestinal, cutaneous and nephrological AEs were far more common in the older subgroup than in the other group [17], overall hinting the need for a better selection of patients candidate for adjuvant immunotherapy. Consistently, in the present study the analysis of the over 75 years old patients compared to the remaining population demonstrated a modest increase in the incidence of total and severe AEs in the elderly subgroup. In the context of adjuvant therapy, this element highlights the importance of multidisciplinary discussion to carefully estimate the risk–benefit profile tailored on patient-specific characteristics. Interestingly, these results seem to not apply to metastatic MM patients as evidenced by Ben-Betzalel et al. in an observational prospective study on 144 patients over 65 in which elder patients (over 80 years old) showed enhanced anti-PD-1 efficacy compared to under 80 years old patients with no significant differences in terms of AEs [18].

Even though DMFS was not available as a result of the present study, due to the low number of events reported, in the high-risk CM adjuvant setting this parameter stands as a critical endpoint, reflecting systemic disease control and improving patients’ quality of life. Nonetheless, it has to be stated that a weak correlation seems to be found between RFS or DMFS and overall survival in the adjuvant setting, as registrational trials and real-world stage III adjuvant studies failed to demonstrated a statistically significant overall survival benefit even in the presence of RFS/DMFS favorable hazard ratio [19]. Finally, as observed in the flowchart enrollment (Supplementary Fig. 1) a number of patients was excluded from the present study due to being unfit for adjuvant immunotherapy or for individual refusal. These patients represent a subgroup of patients not considered in the registrational studies as well as other in observational studies, whose outcomes are unknown and that could affect the efficacy estimates in the present setting. Future studies including these patients could be able to better clarify the real advantages related to anti-PD-1 adjuvant therapy.

Until mature overall survival data become available, no definitive conclusions can be drawn. From the patient’s perspective, however, even delaying recurrence is a meaningful outcome, as it may also enhance opportunities to access novel therapies [18].

## Supplementary Information

Below is the link to the electronic supplementary material.

Supplementary file1 (DOCX 1975 kb)

Supplementary file2 (DOCX 78 kb)

## Data Availability

No datasets were generated or analysed during the current study.
